# Effect of photoperiod and light intensity on learning ability and memory formation of the pond snail *Lymnaea stagnalis*

**DOI:** 10.1007/s10158-020-00251-5

**Published:** 2020-10-19

**Authors:** Ahmed A. A. Hussein, El-Sayed Baz, Janine Mariën, Menerva M. Tadros, Nahla S. El-Shenawy, Joris M. Koene

**Affiliations:** 1 Department of Zoology, Faculty of Science, Suez Canal University, Ismailia, Egypt; 2 Department of Ecological Science, Faculty of Science, Vrije Universiteit, Amsterdam, The Netherlands; 3 Malacology Lab, Theodor Bilharz Research Institute (TBRI), Giza, Egypt

**Keywords:** Conditioning, Gastropod, Light intensity, Memory, MIP II, Mollusk, Photoperiod

## Abstract

Natural light is regarded as a key regulator of biological systems and typically serves as a Zeitgeber for biological rhythms. As a natural abiotic factor, it is recognized to regulate multiple behavioral and physiological processes in animals. Disruption of the natural light regime due to light pollution may result in significant effects on animal learning and memory development. Here, we investigated whether sensitivity to various photoperiods or light intensities had an impact on intermediate-term memory (ITM) and long-term memory (LTM) formation in the pond snail *Lymnaea stagnalis*. We also investigated the change in the gene expression level of molluscan insulin-related peptide II (MIP II) is response to the given light treatments. The results show that the best light condition for proper LTM formation is exposure to a short day (8 h light) and low light intensity (1 and 10 lx). Moreover, the more extreme light conditions (16 h and 24 h light) prevent the formation of both ITM and LTM. We found no change in MIP II expression in any of the light treatments, which may indicate that MIP II is not directly involved in the operant conditioning used here, even though it is known to be involved in learning. The finding that snails did not learn in complete darkness indicates that light is a necessary factor for proper learning and memory formation. Furthermore, dim light enhances both ITM and LTM formation, which suggests that there is an optimum since both no light and too bright light prevented learning and memory. Our findings suggest that the upsurge of artificial day length and/or night light intensity may also negatively impact memory consolidation in the wild.

## Introduction

As an abiotic factor, natural light is regarded as a key regulator of biological systems and generally acts as a Zeitgeber for biological rhythms (Bradshaw and Holzapfel 2010). One of the most common environmental cycles is the day-night cycle which can differ across the seasons depending on the latitude. Even though natural light is not constant, but varies over time in terms of photoperiods and light intensity, this provides sufficient information for entraining biological rhythms (Gorman et al. 2005). Nevertheless, in recent years it has become clearer that the use of artificial light, as part of increased human activity in environments, can affect or even shift the natural rhythmicity of animals (Gaynor et al. 2018). This disruption of the natural light regime is referred to as light pollution and is commonly defined as the change of natural light patterns in the night environment caused by the introduction of artificial light. Hölker and co-workers showed that the use of artificial lighting has been spreading at an average rate of 6% every year (Hölker et al. 2010). This can be in the form of direct exposure of the environment surrounding light sources like street lamps, traffic, greenhouses, and agricultural systems but also through sky glow resulting from such illumination.

There is abundant evidence that reproduction, energy storage, and neuronal activity in animals can be disrupted by changing the information acquired from the natural light–dark cycle (Navara and Nelson 2007). Such an influence on activity may include learning ability and memory formation that is controlled by neuronal circuits. Hence, disruption of the natural light regime may result in significant effects on animals’ learning abilities and memory formation. Since memory and its consolidation play a fundamental role in how animals respond to different life-style choices (Martin et al. 2000), overall behavior may change as a result of disrupted learning and memory and lead to different choices that may result in environmental disruption on the long-term. For instance, some animals change their behavior and tend to preserve energy during times of limited food supply using short-day conditions as a cue, since this generally indicates the beginning of winter (e.g., hamster) (Bilbo and Nelson 2004; Healy et al. 2005). More importantly, an earlier study found that rats exposed to a short day were observed to have specific spatial memory impairments when compared to rats exposed to a long day (Pyter et al. 2005). The changes in spatial memory may be induced by light pollution and can lead to a long-term change in the behavior of animals including: invertebrates, fish, amphibians, reptiles, birds, and mammals (Davies et al. 2014; Gaston et al. 2013; Gaston and Bennie 2014; Gauthreaux Jr et al. 2006; Hölker et al. 2010; Longcore and Rich 2004, 2006), and may have consequences for key biological process (Lewanzik and Voigt 2014).

However, unnatural exposure to light has been shown to deregulate learning and memory processes in vertebrates, much less is known about the effects on invertebrates. Moreover, there is a lack of information about how artificial light can affect learning skills and memory formation. To address this knowledge gap, artificial light should be separated into its relevant components: photoperiods, wavelength (color), and intensity. To disentangle the effects of two of these components, photoperiod, and intensity, while keeping wavelength constant, we here used the pond snail *L. stagnalis* as a model species because it offers several advantages (Fodor et al. 2020). Firstly, this species responds in a highly consistent manner to operant conditioning of aerial respiration, the memory of which has already been shown to be altered by environmentally-relevant stimuli (Lukowiak et al. 1996, 2000, 2010). Secondly, its simple central nervous system (CNS) expresses many genes responsible for the secretion of different hormones, and proteins that are involved in the formation of memory. A prominent one among these genes is the molluscan insulin-related peptide II (MIP II), which has been previously assigned to be involved in the processes of LTM (Azami et al. 2006). Thirdly, while the established breeding and housing conditions for this species involve keeping it under 12 h light and 12 h dark, the current research will also clarify what the best light condition is to perform such a learning experiment. Therefore, in this study, we investigated whether exposure to different photoperiods, and light intensities have an impact on learning, and memory formation, by testing intermediate-term memory (ITM) and long-term memory (LTM) in the pond snail *L. stagnalis*. We combined this with measuring the expression level of the MIP II gene in the CNS of the treated snails.

## Materials and methods

### Animals

*Lymnaea stagnalis* snails were 16 weeks old, with an average shell length of 3.0 ± 0.2 cm, from an age-synchronized population cultured at the Vrije Universiteit Amsterdam, The Netherlands. In the breeding facility, the snails were kept in a circulation system of copper-poor freshwater (average water characteristics: hardness 1.48 mmol/L, pH 8.12, total organic carbon 1.9 mg/L) at 20 ± 1 °C in a 12 h light/12 h dark cycle (broad-spectrum daylight at 1000 ± 100 lx) and fed on broad-leaved lettuce ad libitum.

### Experimental setup

The light treatment took place in a breeding rack setup with five shelves. The light regime of each could be controlled separately via a custom-made dedicated broad-spectrum LED-light strip system and that could each be closed from external, room lighting with a hatch. The LED strip ensured equal lighting across the whole shelf it illuminated. On every shelf, twelve snails were individually housed in a container (with a ground surface of 7.5 cm × 7.5 cm and an opening measuring 9.0 cm × 9.0 cm, with a height of 10.0 cm). For water flow-through purposes, containers were placed in sets of three in a bigger container; every container was labeled with the corresponding light treatment. On every shelf, multiple water taps were available to individually provision each bigger container with water to maintain a constant dripping flow of copper-poor freshwater at 20 ± 1 °C with the same water characteristics mentioned before.

This setup was first used for a photoperiod experiment and subsequently for a light intensity experiment. Each of these experiments lasted for 4 weeks. For the photoperiod experiment, 60 snails were randomly assigned to one of the five shelves where they were exposed to one of the following light regimes: full night (0 h Light: 24 h Dark), short day (6 h Light: 18 h Dark), normal day (12 h Light: 12 h Dark), long day (18 h Light: 6 h Dark), and full day (24 h Light: 0 h Dark). During these light treatments, the light intensity was set to 1000 ± 100 lx, which is equivalent to the breeding facility’s light and corresponds to natural light intensity in the shadow. For the light intensity experiment, a new set of 60 snails was randomly divided over the five shelves. All shelves now had a normal day (12 h Light: 12 h Dark) light cycle that only differed in intensity. The used intensities were 1, 10, 100, and 1000 lx. For both experiments, containers were exchanged with new clean containers every week, and all snails were fed daily with a standard disk of lettuce (19.6 cm^2^) at 10 a.m.

### Learning and memory assay

**Fig. 1:**
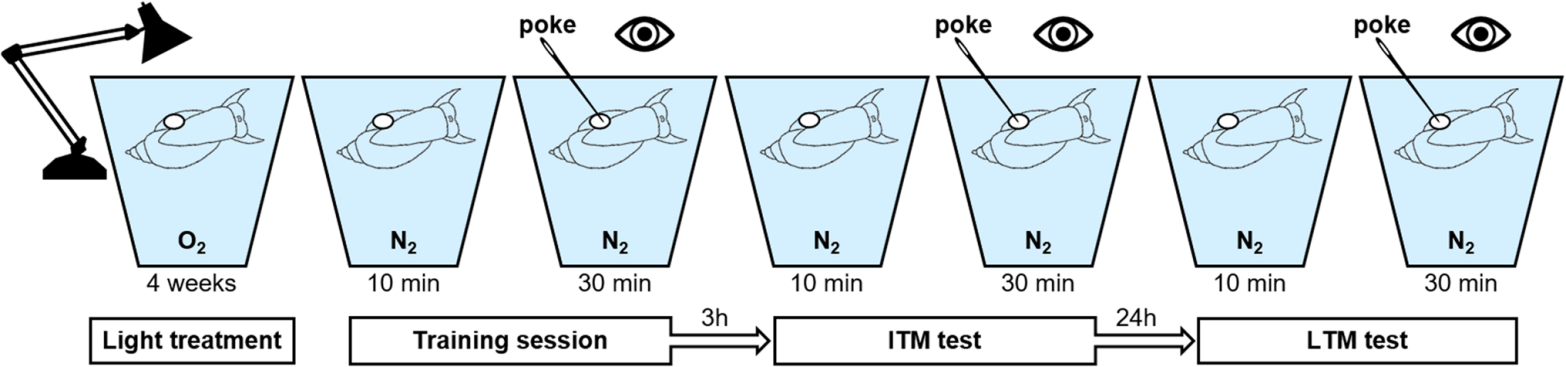
The standard protocol of learning and memory testing for *Lymnaea stagnalis*. For all light treatments, the Zeitgeber time Zero (the time and the light went on) was 6:00 a.m. and the time when the light went off was dependent on the total exposure time. During this 4-week treatment, normal oxygen levels were present (O_2_). All snails were trained once, separately in a small container which was placed in a bigger container with low-oxygen water (N_2_-perfused) that could fit 12 small containers at a time; this was always done at 8:00 a.m. ITM was tested 3 h later at 11:00 a.m. and LTM was tested 24 h later at 8:00 a.m. of the next day. Poke indicates a physical touch to the pneumostome each time a snail attempted to attain aerial respiration. Note that the reading lamp icon is simply used to visually indicate the light exposure and does not reflect the actual LED light strips used

After 4 weeks of exposure to a light regime, for both experiments, we tested intermediate-term memory (ITM) and long-term memory (LTM) formation in 12 treated snails for each light regime. The standard operant conditioning protocol made use of the bimodal breathing behavior of this species to test memory formation and extinction (Lukowiak et al. 1996). Briefly, this makes use of the fact that in highly oxygenated water, *L. stagnalis* absorbs oxygen directly across its body wall from the water, but when dissolved oxygen levels drop it switches to aerial respiration using a respiratory orifice (pneumostome) (Fig. 1). For all light treatments, the zeitgeber time Zero (ZT0; the time the light went on) was 6:00 AM, and the time when the light went off was dependent on the total exposure time. During this 4-week treatment, normal oxygen (O_2_) levels were present. All snails were trained once and separately in a small container which was placed in a bigger container with low-oxygen water (N2-perfused) that could fit 12 small containers at 8:00 a.m. (ZT02).

To maintain low levels of oxygen during the single training session as well as ITM and LTM tests, a strong bubbling of N_2_ through the bigger container was applied for 30-min before sessions. Moreover, to avoid disturbing the animals and to keep the same oxygen levels low during the whole experiment at the same time, the N_2_ bubbling was then reduced and continued at a low-level during acclimation, training, and memory testing. Snails were placed into the hypoxic environment for a 10-min acclimation period, followed by a 30-min period in all training and memory test sessions. During these 30-min sessions, a physical touch (poke) was applied with a sharp wooden stick to the pneumostome each time the snail attempted aerial respiration. Pokes were sufficiently strong to close the opening of the pneumostome and at the same time mild enough so as not to cause full withdrawal response of the snail. The number of pokes (i.e., attempted pneumostome openings) was recorded for each snail over the 0.5 h sessions. To distinguish whether ITM and LTM were formed following the single 0.5 h training session or not, the recorded number of pokes during memory testing was compared with the number in the training session (Parvez et al. 2006; Sangha et al. 2003).

Therefore, we assessed the effect of these light regimes on aerial breathing, learning, ITM, and LTM formation, using the standard protocol (Fig. 1). ITM and LTM were considered present if the number of attempted pneumostome openings (number of pokes) during the ITM test was significantly less than during the training session; furthermore, the LTM response should not be significantly less than the response during ITM (Braun and Lukowiak 2011).

### Gene expression (qPCR)

Following the learning and memory experiments, the snails were sacrificed directly after their LTM test by snap freezing in liquid nitrogen. The CNS of each individual was dissected out and individually stored at − 80 °C. After RNA extraction and the cDNA synthesis, the MIP II gene expression level (*n* = 3 per treatment) was determined via quantitative real-time PCR (qPCR).

RNA isolation was done according to (Pahlevan Kakhki 2014). Briefly, we started with “dry” crushing until the tissue was thawed. Then, Trizol was added to the crushed tissue and pestle in 4 steps of 375 µl (in total 1500 µl). After incubation at room temperature for 5 min, 300-µl chloroform was added and the samples were shaken for a minute by hand. Subsequently, they had centrifuged at 12.000 g at 4 °C for 15 min. The aqueous phase was then transferred to a new 2.0 mL tube after which 1 × volume 2-propanol was added to the sample. The tube was then well shaken and incubated at RT for 10 min after which it was centrifuged again at 12.000 g at 4 °C for 10 min. The complete supernatant was removed very carefully. The remaining pellet was washed with 1500 µl 75% ethanol and centrifuged at 12.000 g at 4 °C for 10 min after which the ethanol was removed. Then, a DNAse treatment was applied to digest any DNA contamination. Finally, phenol and chloroform were added to the sample which was then centrifuged multiple times to precipitate the RNA. The RNA pellet was then washed by 2-propanol and 75% ethanol and then dissolved in 100 µl RNAse-free H_2_O. RNA samples were labeled with “RNA”, a code, and a date, then stored for the short term on ice or in the fridge and if storage was for more than a few hours in a − 80 °C freezer.

**Table 1:** Sequences of the forward and reverse primers of the target and housekeeping genes

Gene	Label	Sequence (5′–3′)	Efficiency (%)
Molluscan insulin-related peptide II	LS-MIP II-F	TGC AGA CCA ACC AGG AAG TT	106.1
	LS-MIP II-R	GGT GAG AAG CAC TGT GAC CAC	
Elongation factor	LS-EF-F	CCACAACTGGCCACTTGATCTAC	92.4
	LS-EF-R	AGGAACCCTTGCCCATCTCTT	
Tubulin	LS-TUB-F	CGAATACCAGCAGTACCAGGATG	91.4
	LS-TUB-R	TTTAGGCATATTCCTGTCCCTCC	

The efficiency percentage indicates the number of copies of the PCR product that doubled in size during the logarithmic stage of the PCR reaction

After RNA isolation, all samples were run on gel-electrophoresis as a quality control step for RNA samples to check for fragments and degradation. The best three samples of each group (*n* = 3) were then chosen for cDNA synthesis.

RNA-concentrations were determined using a NanoDrop Spectrometer ND-1000 with NanoDrop1000 software version 3.7.1 (Thermo Scientific, Wilmington, USA). All samples were diluted to the same RNA concentration in RNase-free MilliQ water. The cDNA reaction was carried out with the M-MLV Reverse Transcriptase Kit (Promega, USA) on a Bio-Rad T100 (Bio-Rad, Hercules, USA). Quantitative PCR was used to determine the relative gene expression of MIP II. Primers for this target gene were chosen according to the recent literature (Azami et al. 2006; Hatakeyama et al. 2013; Murakami et al. 2013). Also, we chose primers for two housekeeping genes, Elongation factor (EF), and Tubulin (TUB). Primers were developed with Primer express 1.5 for, with a melting temperature of 80–100 °C, a primer length between 20 and 25 bp, GC% of the primers 40–45%, and an amplicon length of 90–120 bp (Table 1). In the qPCR-test, the *C*(*t*) values of the experimental MIP II gene were quantified relative to the *C*(*t*) values of the two housekeeping genes. The amount of amplified cDNA was measured with the double strand-binding Sybr green as a fluorescent reporter (SensiMix-SYBR Green, No-ROX Kit, Bioline, USA).

Finally, for all assays the reaction was carried out with hot start at 95 °C for 10 min then 40 cycles (10 s. 95 °C–30 s. 60 °C), and the plate was read after every cycle. After amplification, melting curves were run between 60 °C and 90 °C and the plate read every 0.5 °C to ensure the specificity of PCR. The qPCRs were carried out in 96-wells plates with qPCR Kit (SensiMix-SYBR Green, No-ROX Kit, Bioline, USA) on a CFX 96 qPCR machine (Bio-Rad, USA). The data were analysed and plotted with Bio-Rad CFX manager software.

### Statistical analysis

The statistics were done with GraphPad Prism 8. The data were grouped according to the response during the training sessions, after 3 h and after 24 h within each light treatment, meaning that the tested factor was the formation of memory. After confirming that the data were normally distributed using a Kolmogorov–Smirnov test, we analysed them using Repeated Measures (RM) ANOVAs combined with post hoc Tukey tests for multiple comparisons between treatments using the training session as the normal, control situation. We also compared the responses between the training sessions of each light treatment to test for initial differences before applying the learning protocol using an ANOVA. For all ANOVA tests, the *F*-value, the adjusted degrees of freedom (for numerator dfn and denominator dfd, in subscript), and the significance (*P*) were reported. Moreover, Expression data were log transformed and analysed using an ANOVA with photoperiod or light intensity as the fixed factor.

## Results

The two experiments were used to investigate whether and how light affects learning and memory formation in the pond snail *L. stagnalis*. Intermediate-term memory (ITM, tested after 3 h) and long-term memory (LTM, tested after 24 h) formation were tested in experiments in which snails were either exposed to different photoperiods or different 12-h light intensities. For individuals from both experiments we also quantified gene expression level in the CNS of MIP II, a gene that was proposed to be involved in the LTM formation.

### Naïve aerial respiration behavior

**Fig. 2:**
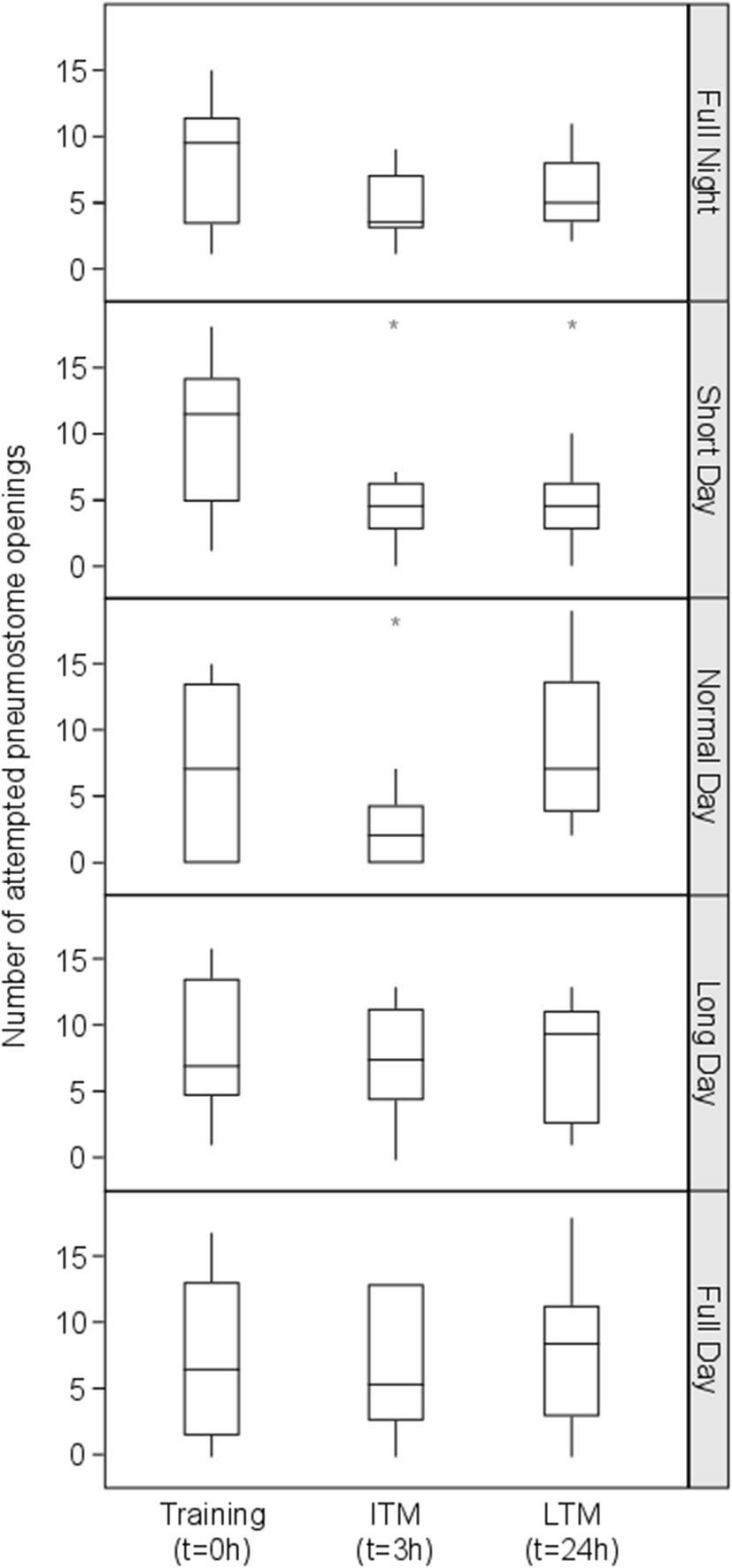
Effect of photoperiod on ITM and LTM. Light treatments are full night (0 h Light: 24 h Dark), short day (6 h Light: 18 h Dark), normal day (12 h Light: 12 h Dark), long day (18 h Light: 6 h Dark), and full day (24 h Light: 0 h Dark) and indicated on the right of each plot. All photoperiods used light of 1000 ± 100 lx. The horizontal lines of each outlier box plot show the median (50th percentile) and quartiles (25th and 75th percentiles) for the number of pokes, with the vertical whiskers indicating the range and open circles indicating outliers. Asterisks (*) indicate a significant difference from Training

**Fig. 3:**
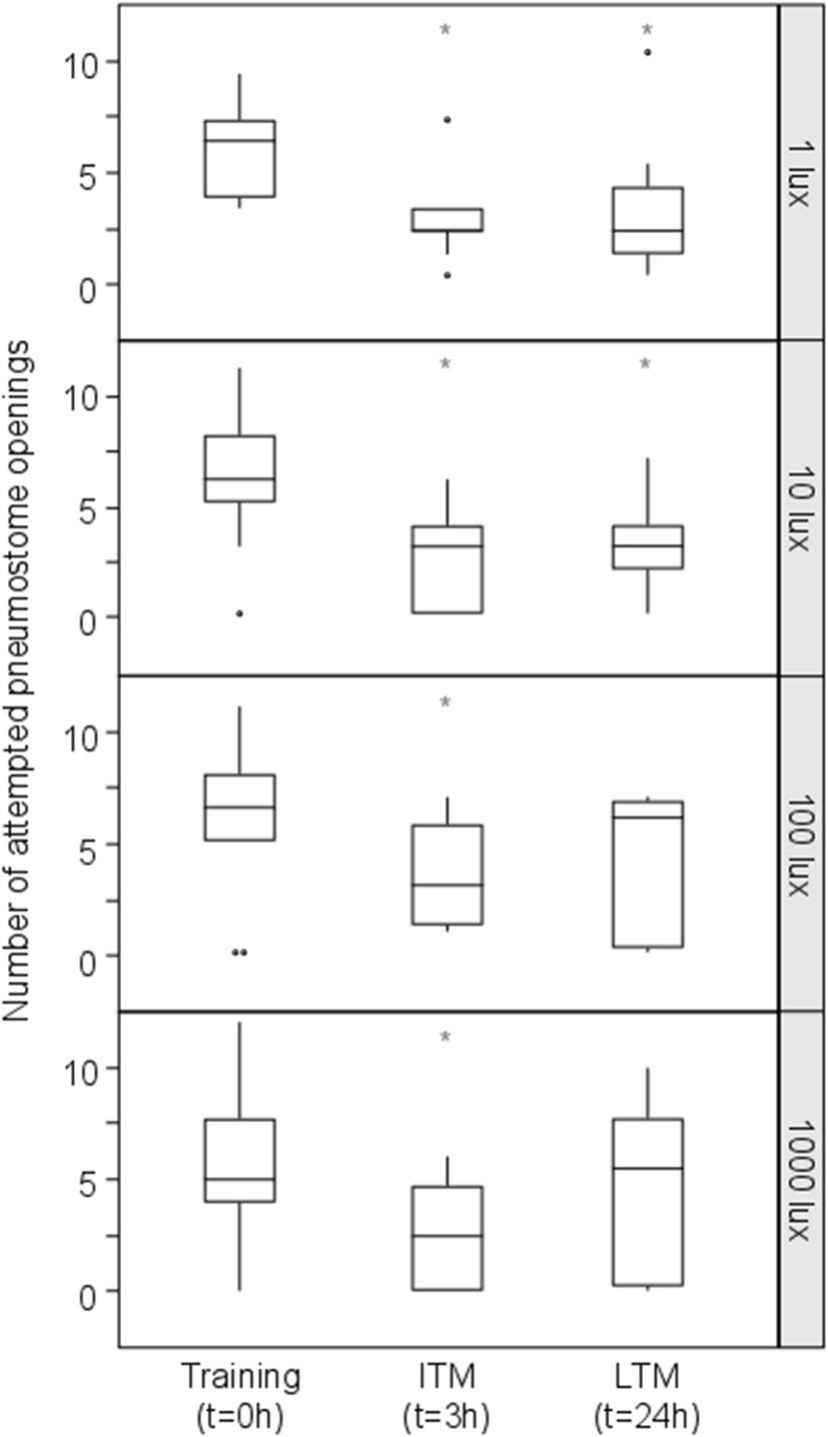
Effect of light intensity on ITM and LTM. Light treatments are the intensity of light, expressed in lux, during the 12 h of light and indicated on the right of each plot. The horizontal lines of each outlier box plot show the median (50th percentile) and quartiles (25th and 75th percentiles) for the number of pokes, with the vertical whiskers indicating the range and open circles indicating outliers. Asterisks (*) indicate a significant difference from Training

To test whether the naïve aerial respiration behavior of the pond snail is photoperiod-specific, before the effect of training, we determined the number of pokes which represents the number of attempts to open their pneumostome for aerial respiration. Our results reveal no statistical difference in the naïve frequency of performing aerial respiration between the five photoperiod treatments (ANOVA: *F*_3.312, 38.92_ = 0.6327, *P* = 0.6153), the compared data can be seen in the left box plot of each graph in Fig. 2. There was also no significant difference between the four light intensity treatments (ANOVA: *F*_2.888,31.77_ = 0.9766, *P* = 0.4135; compare left box plot of each graph in Fig. 3). This indicates that the respiratory behavior itself, which we used in the standard protocol to assess learning, was not already altered by any of the light treatments.

### Photoperiod

To test for the effect of light–dark cycle on memory formation, snails were exposed to different photoperiods with a light intensity set to 1000 ± 100 lx, which mimics the natural light intensity in the shadow during sunshine. Under normal day (12 h light), a single training session resulted in ITM formation after 3 h, but not LTM formation when tested 24 h later (RM ANOVA: *F*_1.520, 13.68_ = 5.183, *P* = 0.0278*r* post hoc Tukey test Training versus ITM and LTM, *P* = 0.0438; *P* = 0.8383, respectively; Fig. 2). Interestingly, for short day (6 h light) both ITM and LTM were formed (RM ANOVA: *F*_1.321, 11.89_ = 12.21, *P* = 0.0028; post hoc Tukey test Training vs. ITM and LTM, *P* = 0.0035; *P* = 0.0232, respectively; Fig. 2). In contrast, long day snails (18 h light) showed no significant changes for both ITM and LTM (RM ANOVA: *F*_2, 18_ = 0.2549, *P* = 0.7778; Fig. 2). Furthermore, for full night (0 h light) or full day (24 h light) we found also no learning effect (RM ANOVA; full night: *F*_1.875, 20.62_ = 3.237, *P* = 0.0626; full day: *F*_2, 18_ = 0.1109, *P* = 0.8956; Fig. 2).

### Light intensity

Since the snails showed no LTM retention under normal day with bright light (~ 1000 lx; Fig. 3), while ITM memory was formed, we decided to test whether the intensity of light was responsible for a lack of LTM formation. The exposure to 12 h of light at different intensities had an overall effect on learning ability of the snails (RM ANOVA; 1 lx: *F*_1.578, 17.36_ = 9.383, *P* = 0.0029; 10 lx: *F*_1.839, 20.23_ = 8.649, *P* = 0.0024; 100 lx: *F*_1.981, 21.79_ = 3.819, *P* = 0.0382; 1000 lx: *F*_1.556, 17.12_ = 3.564, *P* = 0.0504; Fig. 3). Although all snails exposed to 12 h of light at different intensities formed ITM, snails maintained under lower light intensities (i.e., 1 and 10 lx) showed a stronger decrease in the number of attempts to open their pneumostome in comparison to snails maintained under high light intensities (i.e., 100 and 1000 lx) (post hoc Tukey test Training vs. ITM: 1 lx *P* = 0.006; 10 lx *P* = 0.0057; 100 lx *P* = 0.0440; 1000 lx *P* = 0.0420). Furthermore, only snails from treatments that resulted in strong ITM also formed LTM. This was the case for the 1 and 10 lx treatments (post hoc Tukey test Training vs. LTM: *P* = 0.0132 and *P* = 0.0399, respectively; Fig. 3) but not for the 100 and 1000 lx treatments (post hoc Tukey test Training vs. LTM: *P* = 0.3014 and *P* = 0.8224, respectively; Fig. 3).

### MIP II expression

**Fig. 4:**
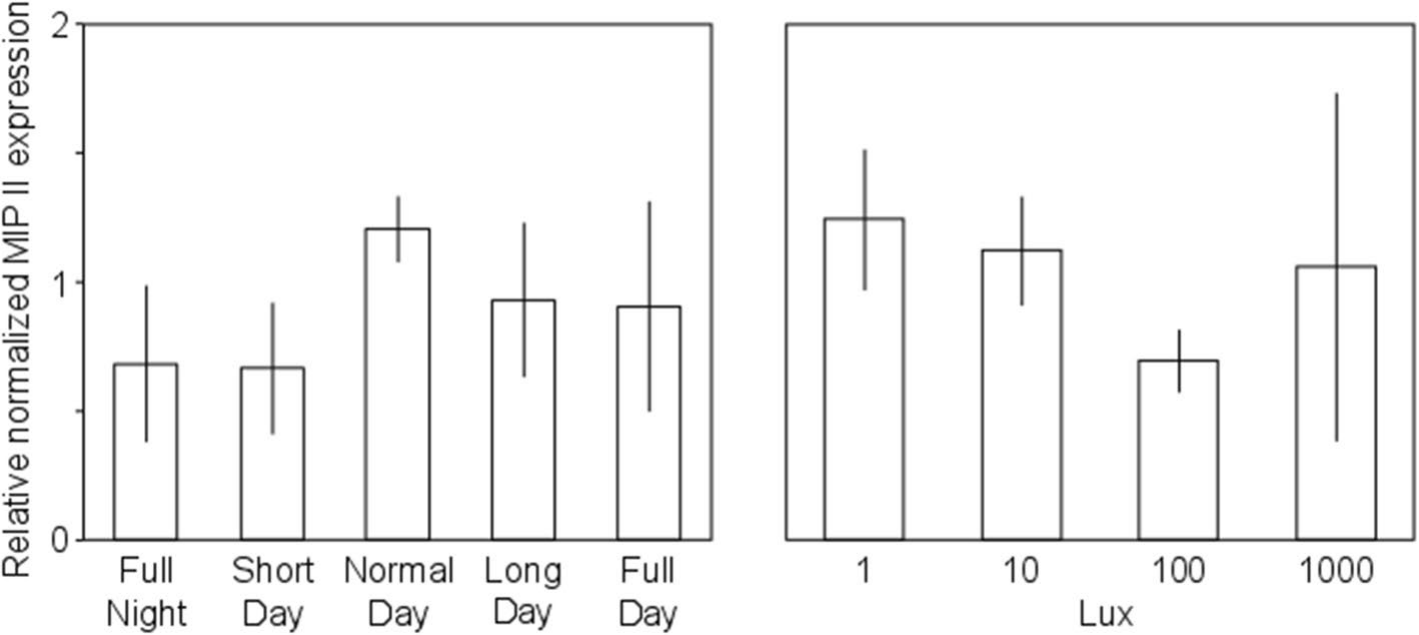
The relative expression level of the MIP II gene between different light treatments. The *x*-axis represents the different light treatments and the *y*-axis represents the gene expression level of MIP II normalized against the housekeeping genes. The bar plots show the expression level of each treatment with SE

The role of MIP II gene expression in aerial respiration LTM formation using qPCR was examined. The expression levels of MIP II for each individual (*n* = 3 per treatment) were normalized against the corresponding expression level of the housekeeping genes EF and TUB. For photoperiod and light intensity experiments, there was no significant difference in the relative expression level of MIP II between treatments (Fig. 4).

## Discussion

Prior studies have shown that artificial night lighting affects the behavioral and physiological functioning of aquatic and terrestrial animals including invertebrates (Davies et al. 2012), amphibians (Buchanan 2006), fishes (Nightingale et al. 2006), birds (Gauthreaux Jr et al. 2006), and humans (Beier 2006). Among other things, such light conditions can positively or negatively modify the acquisition or consolidation of memories (Chellappa et al. 2014; LeGates et al. 2012; Shan et al. 2015). Hence, we hypothesized that disrupting the light–dark cycle of the pond snail *L. stagnalis*, via exposure to different photoperiods and different light intensities, would have an impact on learning and memory formation at behavioral and molecular levels in this naturally diurnal species. To assess learning, the operant conditioning training of aerial respiration that is an established technique in this species was used (Lukowiak et al. 1996). We did not find changes in naïve aerial respiratory behavior in response to the different light regimes per se. However, we did observe that light conditions play an important role in the formation of memory in *L. stagnalis* after training.

When testing for photoperiod-dependent memory after single-session training, the complete absence of light cues (i.e., full night), as well as excessive exposure to light (full day), resulted in impairment of both ITM and LTM formation. Therefore, pond snails failed to learn in the absence of a light–dark cycle within our experimental setup. When a light–dark cycle was present, our data show that a short photoperiod (short day) resulted in ITM and LTM formation, but not in the longer photoperiods (normal day and long day). This indicates that longer exposure to light impairs the formation and/or retrieval of LTM and suggests that it varies circannual. Importantly, we can conclude that the pond snail’s ability to learn, consolidate, and maintain memory may be impaired in habitats where they are exposed to a bright artificial nightlight. This finding is consistent with previous reports suggesting that photoperiod influences circadian activity, learning, and memory in mammals (Dellapolla et al. 2017; LeGates et al. 2012; Loh et al. 2010; Ma et al. 2007; MacDonald et al. 2007; Smarr et al. 2014). Moreover, a recent study using *Drosophila melanogaster* also has emphasized that disrupting its light–dark cycle effect LTM (Inami et al. 2020).

In the second experiment, where snails were maintained under a normal day photoperiod, the effect of different light intensities on memory formation was examined. All treatments showed ITM, which agrees with the normal day treatment group of the previous experiment. Moreover, LTM did not form at the higher light intensities. These results support the idea that the effects of light on memory also depend on the light’s intensity because the formation of ITM in snails kept under lower light intensities (i.e., 1 and 10 lx for 12 h) seemed stronger in comparison to snails maintained under higher light intensities (i.e., 100 and 1000 lx for 12 h). Also, LTM was only maintained under the lower light intensities, while LTM seemed to be disrupted under the higher light intensities used. The lack of LTM formation might indicate that snails were stressed as a result of excessive light exposure, which could lead to repression of synaptic morphology and altered neurotransmitter levels necessary to form LTM. Indeed, it has been shown that high levels of stress can block memory processes in this pond snail species (Lukowiak et al. 2014) as well as in mammals (Kim and Diamond 2002; McEwen 1999). The present data are consistent with the notion that high intensities of light can act as stressors, although the mechanisms by which light influences or controls cellular function remain to be fully revealed.

Finally, we investigated whether the gene expression of molluscan insulin-related peptide II (MIP II) in the CNS changed after aerial respiration LTM conditioning in different light treatments. This gene was chosen for *L. stagnalis* because it was shown that MIP II expression was upregulated during LTM formation in conditioned taste aversion training (Azami et al. 2006), and that MIP II was expressed in the cerebral ganglia (Meester et al. 1992). Many studies indicate that insulin and insulin-like peptides are involved in the processes of LTM formation in both invertebrate and rodents (Chambers et al. 2015; Dou et al. 2005; Kojima et al. 2015; Kukushkin et al. 2019; Lin et al. 2010; Murakami et al. 2005; Ramsey et al. 2005; Zhao et al. 2019). This is supported by findings that the formation of LTM was accompanied by changes in the synaptic morphology (Geinisman 2000; Kawai et al. 2002). Moreover, neurite formation is partly controlled by MIP II, so upregulation seems needed to initiate alterations in synaptic morphology that are associated with memory formation and its maintenance (Azami et al. 2006; Murakami et al. 2013). Nevertheless, we did not detect any significant differences in the expression level of MIP II in those treatments that showed LTM (low light intensity and shorter photoperiod) in our current data. Although these findings are based on a relatively small but standard qPCR sample size, they might still indicate that MIP II is not involved in LTM formation via operant conditioning using aerial respiration. If this is confirmed, for example in a follow-up study using a larger qPCR sample size, this might indicate that LTM is formed via a different route that does not require the production of MIP II, in contrast to LTM produced by conditioned taste aversion (Azami et al. 2006). LTM seems to require protein synthesis; however, recent work on aversive olfactory conditioning in *D. melanogaster* has shown that LTM produced by a single-trial training can be formed immediately without the requirement for protein synthesis-dependent consolidation (Zhao et al. 2019). Likewise, studies on *Caenorhabditis elegans* and *Aplysia californica* showed that LTM can be formed both dependent and independent of protein synthesis (Chambers et al. 2015; Conte et al. 2017; Stein and Murphy 2012; Timbers and Rankin 2009). Hence, further studies will be needed to identify the cellular and molecular mechanisms that act as regulators of neuronal plasticity during aerial respiration LTM formation.

In summary, we demonstrated for the first time that photoperiod and light intensity are important factors for the appropriate acquisition as well as the consolidation of memory in *L. stagnalis*. Also, the learning and memory formation processes for operant conditioning using aerial respiration were sensitive to both photoperiod and light intensity. Low light intensity and a relatively short-day length improved learning ability. Moreover, the finding that MIP II gene expression did not seem to change even though LTM formed warrants further investigation, for instance, by increasing sample size or testing another pathway. Interestingly, while for breeding and maintenance purposes of *L. stagnalis* a normal 12L:12D regime is standard, our findings indicate that this light condition may not be ideal for the operant conditioning learning task used here and warrants testing whether other types of associative learning procedures are affected similarly. Finally, in general our results clearly indicate that light conditions can impacts learning and memory and that these abilities also need to be considered in a light pollution context.
